# Antigenicity of *Leishmania*-Activated C-Kinase Antigen (LACK) in Human Peripheral Blood Mononuclear Cells, and Protective Effect of Prime-Boost Vaccination With pCI-neo-LACK Plus Attenuated LACK-Expressing Vaccinia Viruses in Hamsters

**DOI:** 10.3389/fimmu.2018.00843

**Published:** 2018-04-23

**Authors:** Laura Fernández, Eugenia Carrillo, Lucas Sánchez-Sampedro, Carmen Sánchez, Ana Victoria Ibarra-Meneses, Mͣ Angeles Jimenez, Valter dos Anjos Almeida, Mariano Esteban, Javier Moreno

**Affiliations:** ^1^WHO Collaborating Center for Leishmaniasis, National Center of Microbiology, Instituto de Salud Carlos III, Madrid, Spain; ^2^Department of Molecular and Cellular Biology, Centro Nacional de Biotecnología, Consejo Superior de Investigaciones Científicas (CNB-CSIC), Madrid, Spain; ^3^Departamento Medicina y Cirugia Animal, Facultad de Veterinaria, Universidad Complutense de Madrid, Madrid, Spain; ^4^Instituto Gonçalo Moniz, Fundação Oswaldo Cruz, Salvador, Bahia, Brazil

**Keywords:** vaccine, visceral leishmaniasis, *Leishmania*-activated C-kinase antigen, antigenicity, cytokines, hamster

## Abstract

*Leishmania*-activated C-kinase antigen (LACK) is a highly conserved protein among *Leishmania* species and is considered a viable vaccine candidate for human leishmaniasis. In animal models, prime-boost vaccination with LACK-expressing plasmids plus attenuated vaccinia viruses (modified vaccinia Ankara [MVA] and mutant M65) expressing LACK, has been shown to protect against cutaneous leishmaniasis (CL). Further, LACK demonstrated to induce the production of protective cytokines in patients with active CL or cured visceral leishmaniasis, as well as in asymptomatic individuals from endemic areas. However, whether LACK is capable to trigger cytokine release by peripheral blood mononuclear cells from patients cured of CL due to *Leishmania infantum* (*L. infantum*) or induce protection in *L. infantum*-infected hamsters [visceral leishmaniasis (VL) model], has not yet been analyzed. The present work examines the *ex vivo* immunogenicity of LACK in cured VL and CL patients, and asymptomatic subjects from an *L. infantum* area. It also evaluates the vaccine potential of LACK against *L. infantum* infection in hamsters, in a protocol of priming with plasmid pCI-neo-LACK (DNA-LACK) followed by a booster with the poxvirus vectors MVA-LACK or M65-LACK. LACK-stimulated PBMC from both asymptomatic and cured subjects responded by producing IFN-γ, TNF-α, and granzyme B (Th1-type response). Further, 78% of PBMC samples that responded to soluble *Leishmania* antigen showed IFN-γ secretion following stimulation with LACK. In hamsters, the protocol of DNA-LACK prime/MVA-LACK or M65-LACK virus boost vaccination significantly reduced the amount of *Leishmania* DNA in the liver and bone marrow, with no differences recorded between the use of MVA or M65 virus vector options. In summary, the Th1-type and cytotoxic responses elicited by LACK in PBMC from human subjects infected with *L. infantum*, and the parasite protective effect of prime/boost vaccination in hamsters with DNA-LACK/MVA-LACK and DNA-LACK/M65-LACK, revealed the significance of LACK in activating human and hamster immune responses and support LACK to be a valuable candidate for inclusion in a vaccine against human VL.

## Introduction

Leishmaniasis is one of the most neglected tropical diseases and has strong links with poverty (1). Visceral leishmaniasis (VL) is the most severe form; its annual incidence is 200,000–400,000 cases worldwide, and without treatment mortality is high (2). In South Asia and East Africa, VL is caused by *Leishmania donovani*, while in the Mediterranean, the Middle East and Latin America, the causal agent is *Leishmania infantum (L. infantum)*.

Visceral leishmaniasis treatment is based on pentavalent antimonials, oral miltefosine, liposomal amphotericin B, and paramomycin, but these have been associated with severe toxic side effects and increasing parasite resistance (3). The treatment of leishmaniasis is expensive ranging from US$ 30 to 1,500 for medication alone, which may further increase the poverty of affected individuals (1). While vaccination is the most cost-effective way of controlling infectious diseases (4), no vaccine against human VL exists. However, there are reasons for optimism that one or more safe and effective vaccines against leishmaniasis might be developed (3, 5, 6). A number of parasite antigens have been identified as candidates for vaccine development (5), including *Leishmania* analog receptor for activated C kinase (LACK) (7). *Leishmania*-activated C-kinase antigen (LACK) is a 36 kDa protein highly conserved across *Leishmania* species, and is expressed by both promastigotes and amastigotes (8). LACK antigen from *Leishmania braziliensis, Leishmania guyanensis*, and *Leishmania amazonensis* induces the production of IFN-γ and IL-10 in peripheral blood mononuclear cells (PBMC) from patients with cutaneous leishmaniasis (CL), and IL-10 in those of naïve individuals (7, 9–12). LACK drives the expansion of IL-4 secreting T cells (13), and for that reason LACK vaccination trials used approaches, like cytokines or DNA vectors, to redirect early IL-4 responses to a protective Th1 response (14, 15). In this sense, vaccinia virus is a strong adjuvant and delivery vector altogether, modifying IL-4 secretion of Vβ4 Vα8 CD4+ T cells (16).

Immunization with LACK-expressing plasmid DNA, with altered LACK peptides, or with the purified protein in the presence of IL-12, results in protection against CL in a mouse model, advancing the candidacy of LACK in the production of a vaccine against leishmaniasis (9). Heterologous immunization, i.e., priming with a DNA vector expressing LACK followed by a boost with non-replicating modified vaccinia virus Ankara (MVA) expressing LACK has proven to be an effective protocol in affording protection against *Leishmania major* infection in a murine model of CL, and against *L. infantum* infection in a canine model of VL (17, 18). Further, boosting with LACK-expressing replicative M65 vaccinia virus has been reported to protect against CL in a murine model (17). The protection conferred by heterologous prime/boost immunization involving a DNA vector expressing LACK, plus LACK-expressing M65 virus, however, has not been tested in any experimental model of VL. LACK-expressing M65 and MVA have both been reported to activate polyfunctional CD4+ and CD8+ T cells with effector memory phenotypes. However, prime/boost immunization involving a LACK-expressing pCI-neo plasmid plus LACK-expressing M65 preferentially induced CD4+ T cell responses, while the same plasmid plus LACK-expressing MVA preferentially induced a CD8+ T cell response (17). The replication competence of these viruses has also been described to play an important role in disease prevention (17). The preferential CD4+ T cell response of LACK-expressing M65 plus its replication competence suggest this virus, in combination with a priming LACK-expressing plasmid, might induce potent protection against VL.

With the eventual goal of producing an effective vaccine against human VL caused by *L. infantum*, the aim of this work was to examine the cellular immune response to the LACK protein induced in humans who have been in contact with *L. infantum*, and to assess the parasite efficacy of prime/boost immunization involving a LACK-expressing plasmid plus LACK-expressing MVA or M65 viruses in a hamster model of VL.

## Materials and Methods

### Ethics Statement

Work involving human subjects was approved by the *Hospital de Fuenlabrada* Ethics and Research Committee (APR 12-65 and APR 14-64). All participants gave their written, informed consent to be included. Work involving animals was approved by the Research Ethics and Animal Welfare Committee of the *Instituto de Salud Carlos III*, and performed adhering to Spanish legislation on the protection of animals used for experimentation and other scientific purposes (Royal Decree 1201/2005 and Law 32/2007; this law is a transposition of Directive 86/609/EEC). One efficacy trial was performed in this study, and duplication of the animal experiments was not approved for the Research Ethics and Animal Welfare Committee.

### Study Subjects

Peripheral blood samples were collected between 2013 and 2015 from 13 patients cured of VL (CVL) and 10 cured of CL (CCL) (with cure confirmed at 6 months after the end of treatment). The patients with VL had been treated with liposomal amphotericin B, and those with CL with meglumine antimoniate. All were attended to at the *Hospital de Fuenlabrada*, Madrid. Blood samples were also collected from 90 healthy blood donors at the hospital blood bank. Among these, 18 showed a *Leishmania*-specific cell proliferative response when PBMC were stimulated *in vitro* with soluble *Leishmania* antigen (SLA) (SI > 2.1); these were defined as asymptomatic (ASYMP) (19) and selected for inclusion. Another 14 donors who showed no *Leishmania*-specific cell response were randomly selected for inclusion as endemic-area healthy controls (EC). Other classical assays, such as the leishmanin skin test (LST; no GMP commercial production approved in EU) and the SLA-based ELISA (very low sensitivity for *L. infantum* asymptomatic subjects), were not considered in this study for identification of asymptomatic infections. All patients plus the ASYMP and EC subjects lived in Fuenlabrada, a *L. infantum* post-outbreak area in Madrid (Spain).

### Animals and Parasite Strain

Thirty two, 12-week-old, male golden hamsters (*Mesocricetus auratus*) were purchased from Janvier (France). All were housed in the animal facilities of the National Centre for Microbiology and randomly assigned to one of four experimental groups (see below).

*Leishmania infantum* promastigotes (MCAN/ES/98/LLM-724, JPC strain) were grown for 2 weeks in Novy-MacNeal-Nicolle (NNN) medium and RPMI medium (Gibco, UK) supplemented with 100 UI/ml of penicillin, 100 mg/ml of streptomycin, 2 mM l-glutamine, 2-mercaptoethanol, and 10% heat inactivated fetal calf serum (FCS) (Lonza, Spain).

### Cells, Plasmids, and Viruses

The mammalian expression plasmid vector pCI-neo-LACK has been previously described (8). The empty pCI-neo plasmid (Promega, USA) was used as a control (plasmid-φ).

The viruses used in this study included the MVA strain used as a control (MVA-φ) and recombinant MVA and M65 vaccinia virus strains expressing the *L. infantum* LACK antigen at the viral HA locus (MVA-LACK and M65-LACK, respectively). MVA-LACK and M65-LACK were obtained by transfection with the pHLZ-LACK plasmid, produced by cloning the *L. infantum* LACK gene into the SmaI site of the pHLZ VACV insertion plasmid under the control of the synthetic early/late pE/L viral promoter and the hemagglutinin (HA) flanking sites. Transfection was achieved by infecting pHLZ-LACK plasmid-containing BSC-40 cells (for M65-LACK) or chick cells (for MVA-LACK) with MVA or M65 viruses and harvesting 48–72 h post-infection. Since pHLZ-LACK contains the *Escherichia coli* (*E. coli*) β-glucuronidase gene under the control of the p7.5 early/late viral promoter, β-glucuronidase-producing plaques were identified after the addition of X-Gluc to the agar. Recombinant viruses were plaque purified several times as previously described (20) (checked by PCR), grown in primary chicken embryo fibroblasts (CEF) (from pathogen-free 11-day-old eggs [Intervet, Spain]) in Dulbecco’s modified Eagle’s medium (DMEM) supplemented with 10% FCS in a humidified 5% CO_2_ atmosphere at 37°C and purified by sedimentation after two sucrose-cushions. Viruses were titrated by immunostaining in CEF cells.

For inoculation in animals, pCI-neo-LACK plasmids and LACK-expressing viruses were diluted in endotoxin-free PBS.

### Soluble *Leishmania* and LACK Antigens

Soluble *Leishmania* antigen was prepared from *L. infantum* promastigotes in the stationary phase of growth (MCAN/ES/98/LLM-724, JPC strain). Parasite cultures were centrifuged at 1,000 *g* for 20 min at 4°C. The pellet was then resuspended in lysis buffer (50 mM Tris/5 mM EDTA/HCl, pH 7), subjected to three cycles of freezing–thawing, sonicated, and further centrifuged at 27,000 *g* for 4 h at 4°C. The resulting supernatant was divided into aliquots and stored at −20°C. The protein concentration was determined using the Pierce BCA Protein Assay Kit (Thermo Scientific, USA) following the manufacturer’s recommendations.

Recombinant *L. infantum* LACK was expressed in *E. coli* BL21 pLysS (transformed with plasmid pRSET-B-LACK) as a fusion protein with an N-terminal histidine tag, and purified by affinity chromatography in a Ni^2+^ column. All bacteria were grown in the presence of ampicillin and LACK expression induced with isopropyl thio-β-D-galactoside (final concentration 0.5 mM). Cultures were centrifuged at 3,600 *g* for 15 min, resuspended in lysis buffer (8 M urea, 50 mM Tris-HCL pH 8, 500 mM NaCl), and incubated for 1 h with end-over-end mixing in a tube rotator at 4°C. After a further 30 min centrifugation at 14,000 *g*, the supernatant was incubated with 2 ml of equilibrated ProBond Nickel-Chelating Resin (Invitrogen, USA). The beads were then washed three times with wash buffer (8 M urea, 50 mM Tris-HCL pH 8, 500 mM NaCl, 10 mM imidazol) and elution performed using an increasing imidazol series (final concentration 200 mM). LACK protein for *in vitro* studies was kept in saline in PD10 Desalting Columns (GE Healthcare, USA). LPS levels were determined by the Limulus Amebocyte Lysate kit (Sigma-Aldrich, Heidelberg, Germany), and levels were equal to 0.2 endotoxin units per mg protein.

### Quantification of Cytokines and Granzyme B in Supernatants From PBMC Stimulated With SLA and LACK

Peripheral blood mononuclear cells from study subjects were isolated from heparinized blood in a Ficoll–Hypaque gradient (Rafer, UK) and resuspended in RPMI 1640 supplemented with 10% FCS and 100 U/ml penicillin/streptomycin (Lonza, Sweden) as previously described (21). 2 × 10^5^ cells/well were then distributed into 96-well plates and cultured for 5 days with supplemented RPMI 1640 medium either alone or with 10 µg/ml SLA or 25 µg/ml LACK at 37°C in a 5% CO_2_ atmosphere. The supernatants were then collected and stored at −20°C until analyzed. IFN-γ, TNF-α, granzyme B, and IL-10 were quantified by flow cytometry using the BD Cytometric Bead Array Human Flex Kit (Beckton & Dickinson Bioscience, USA) as previously described (21). Data were analyzed using FCAP Array software v.3.0 (Beckton & Dickinson Bioscience, USA).

### Immunization and Experimental Infection of Hamsters

Hamsters were randomly distributed into four experimental groups of eight animals each: (1) C-PBS—non-vaccinated controls; (2) C-DNA—prime/boost vaccinated with plasmid-φ and MVA-φ; (3) MVA-LACK—prime/boost vaccinated with pCI-neo-LACK and MVA-LACK; (4) M65-LACK—prime/boost vaccinated with pCI-neo-LACK and M65-LACK.

The above priming injections were performed intramuscularly using 100 µg of plasmid-φ or pCI-neo-LACK as required in 100 µl final volumes of PBS. Four weeks later, the animals were boosted intramuscularly (2 × 10^7^ PFU/hamster) with MVA-φ, MVA-LACK, or M65-LACK as required. All immunization protocols were repeated 4 weeks after the initial boost. The C-PBS control group was injected with 100 µl of PBS at each immunization point. Four weeks after the last reinforcement, all hamsters were inoculated with 2 × 10^7^ promastigotes *via* the intracardiac route (22), and 4 months later anesthetized with isoflurane and sacrificed by cardiac puncture.

### PBMC Isolation and Proliferation Assay in Hamsters

Blood was drawn from the hamsters at the time of sacrifice, and PBMC isolated using a Ficoll–Hypaque density gradient. 1 × 10^5^ cells/well were plated on RPMI medium either alone or with 10 µg/ml SLA or 25 µg/ml LACK for 5 days. 5-bromo-2 deoxyuridine (BrdU) (25 µl, 10 µM) was added to each well for the last 18 h to examine lymphocyte proliferation using the BrdU Cell Proliferation Assay Kit (GE Healthcare Life Sciences, UK) according to the manufacturer’s instructions. Results were expressed as stimulation indices, which represent the ratio between the mean absorbance of stimulated cells and that of unstimulated cells (22).

### Immunoenzyme Assay

An aliquot of blood (with heparin) was also taken at the time of sacrifice to provide plasma samples *via* centrifugation at 2,000 *g* for 10 min. The plasma collected was then stored at −20°C until use. Maxisorp microtiter plates (Nunc, Denmark) were antigen-coated overnight with SLA (10 µg/ml) or LACK (25 µg/ml) in carbonate buffer (1 mM Na_2_CO_3_, 28 mM NaHCO_3_, pH9.6) and blocked for 1 h at 37°C with 200 mL of 1% BSA and 0.1% Tween 20 in PBS. PBS containing 0.01% Tween 20 was used to wash the plates three times, which were then incubated for 30 min with 100 µl of plasma diluted 1:100 in buffer (0.1% BSA and 0.1% Tween 20 in PBS). Plates were washed and incubated for 30 min with 1:2,000 horseradish peroxidase-conjugated goat anti-hamster IgG (Abd Serotec, UK) for LACK ELISA, and 1:5,000 for SLA ELISA. o-Phenylenediamine dihydrochloride tablets (Sigma, Spain) were used as the enzyme’s substrate; the reaction was stopped with 50 µl of 1 M H_2_SO_4_. The absorbance was measured at 492 nm in a Multiskan FC microplate photometer (Thermo Scientific, USA).

### Histopathology

All 32 hamsters underwent a complete necropsy and samples of liver, spleen, kidney, and bone marrow were collected from each. Tissues were fixed in 10% buffered formalin, trimmed, processed, and embedded in paraffin wax following routine laboratory procedures, sectioned at 4 µm, and stained with hematoxylin-eosin for histopathological examination under the light microscope. Two samples of liver from the middle and lateral were processed. Two step sections 20 µm apart were obtained for examination from each liver sample. Similar step sections 20 µm apart from spleen, bone marrow, and kidneys were examined for each animal by a trained pathologist blinded for the experiment. Inflammatory and degenerative lesions in each tissue were qualitatively described and scored semi-quantitatively according to their severity as either non-existent (0), mild (+), moderate (++), or severe (+++). Mild was established when inflammatory infiltrates occupied less than 20% of the sections, moderate when inflammatory infiltrates occupied between 20 and 40% of the sections, and severe when inflammatory infiltrates occupied over 40% of the examined parenchyma. Additional specific features such as giant multinucleated cells and Schauman bodies were descriptively noted and recorded for each case.

### DNA Isolation and Quantitative Real Time PCR

During necropsy, liver and spleen samples were homogenized in RPMI medium using a 40 µm stainless steel tissue grinder, and 1 × 10^6^ cells used for total DNA isolation *via* traditional phenol/chloroform extraction and ethanol precipitation. Total DNA was resuspended in 100 µl of distilled water and quantified using a ND-1000 UV-V spectrophotometer (NanoDrop Technology, USA). *Leishmania* DNA was quantified by quantitative real-time PCR (qPCR) using a LightCycler high speed thermocycler and the LightCycler FastStart DNA Master SYBR Green I kit (Roche Diagnostics, Spain), as previously described (22).

### Statistical Analysis

Data were analyzed using the Mann–Whitney *U*-test. Significance was set at *p* ≤ 0.05. The cut-off values for IFN-γ production after incubation with SLA or LACK were determined by calculating the area under the receiver operating characteristic (ROC) curve (AUC) along with the 95% confidence interval (CI). In the figures of the efficacy trial, each dot represents a single hamster, and the black line shows the group geometric mean. All calculations were performed using GraphPad Prism 7.0 software (GraphPad Software, USA).

## Results

### LACK Induced a Significant Increase in TNF-α, IFN-γ, and Granzyme B Production in PBMC From *L. infantum* Cured and Asymptomatic Human Subjects

**Figure 1:**
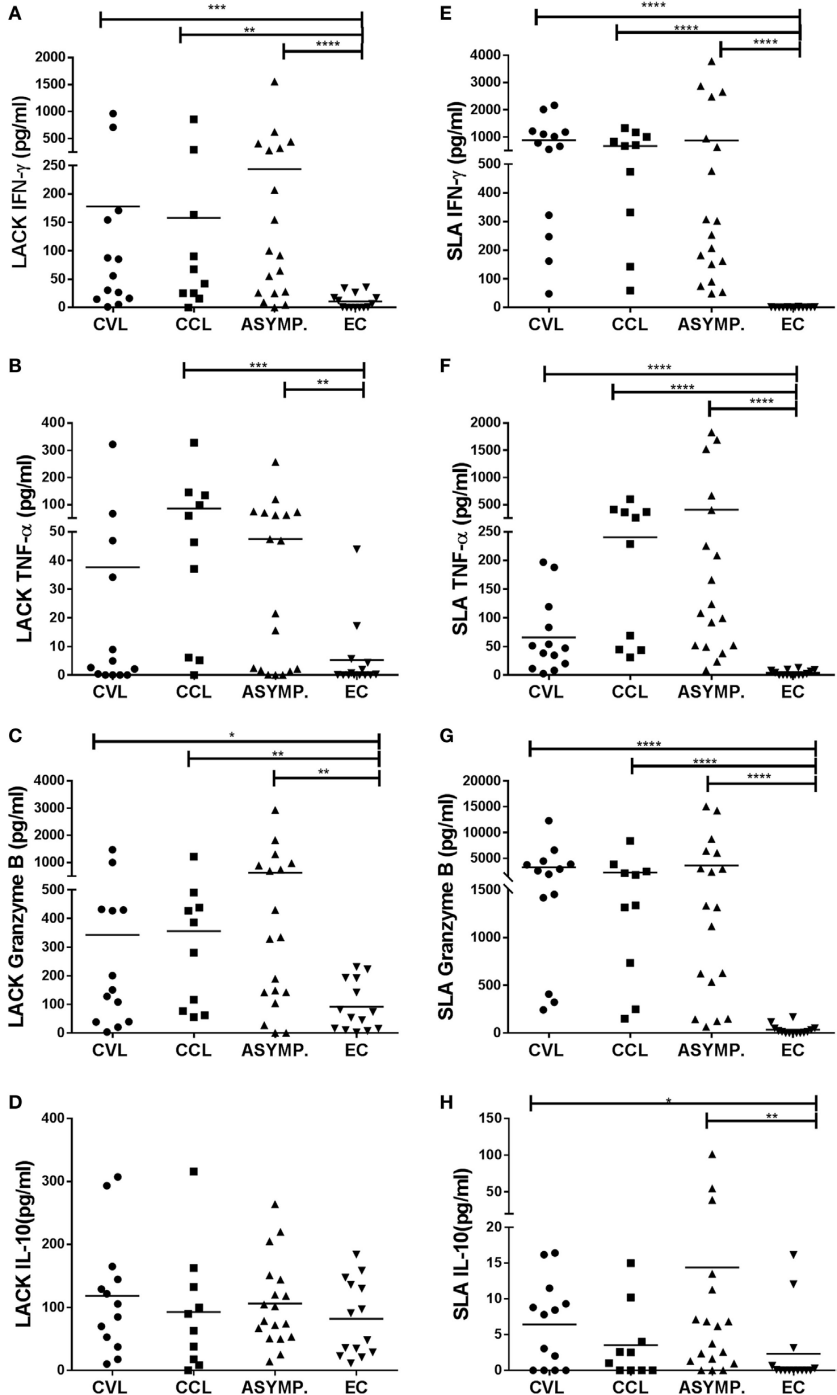
Production of IFN-γ, TNF-α, granzyme B, and IL-10 in peripheral blood mononuclear cells stimulated with *Leishmania*-activated C-kinase antigen [**(A–D)** respectively], and with soluble *Leishmania* antigen [**(E–H)** respectively], in healthy controls (EC, *n* = 14), asymptomatic subjects (ASYMP, *n* = 18), patients cured of cutaneous leishmaniasis (CCL, *n* = 10), and patients cured of visceral leishmaniasis (CVL, *n* = 13). *p ≤ 0.05, **p ≤ 0.01, ***p ≤ 0.001, ****p ≤ 0.0001.

*Leishmania*-activated C-kinase antigen induced a significant increase in the secretion of IFN-γ and granzyme B by the PBMC from the CVL (*p* ≤ 0.001), CCL (*p* ≤ 0.01), and ASYMP subjects (*p* ≤ 0.0001) over that produced by the EC subjects (Figures 1A,C). TNF-α production induced by LACK was significantly increased in the CCL (*p* ≤ 0.001) and ASYMP subjects (*p* < 0.01) (Figure 1B). SLA stimulation led to significant increases in the production of IFN-γ, granzyme B, and TNF-α in the CVL, CCL, and ASYMP subjects (*p* ≤ 0.0001 in all cases) (Figures 1E–G). IL-10 production was detected in all groups, including the controls, after LACK stimulation (Figure 1D). After SLA stimulation, PBMC from the CVL and asymptomatic subjects showed significantly greater IL-10 production compared to the EC subjects’ cells (although levels were low) (Figure 1H).

### LACK Was Recognized by a High Percentage of CVL, CCL, and Asymptomatic Subjects

Following SLA stimulation, the PBMC of all CVL, CCL, and ASYMP subjects showed increased production of IFN-γ compared to the EC subjects’ cells. This cytokine was, therefore, chosen to determine the percentage of individuals that recognized LACK. IFN-γ was produced—and, therefore, LACK recognized—by 69% (9/13) of the CVL subjects, 80% of the CCL subjects (8/10), and 83% of the ASYMP subjects (15/18) (Table 1).

**Table 1:** Percentage of individuals with immune cellular memory against *L. infantum* who recognized *Leishmania*-activated C-kinase antigen and soluble *Leishmania* antigen.

Antigen	Cut-off	Se. (%)	Sp. (%)	AUC	% Recog. CVL	% Recog. CCL	% Recog. Asymp
LACK	21.17	78.05	78.5	0.85	69	80	83
SLA	25.44	100	100	1.00	100	100	100

*Se, sensitivity; Sp., specificity; AUC, area under the curve; CVL, cured of visceral leishmaniasis; CCL, cured of cutaneous leishmaniasis; Asymp, asymptomatic individuals*.

### Hamsters Vaccinated With pCI-neo-LACK/MVA-LACK Had the Highest Anti-LACK IgG Titers

**Figure 2:**
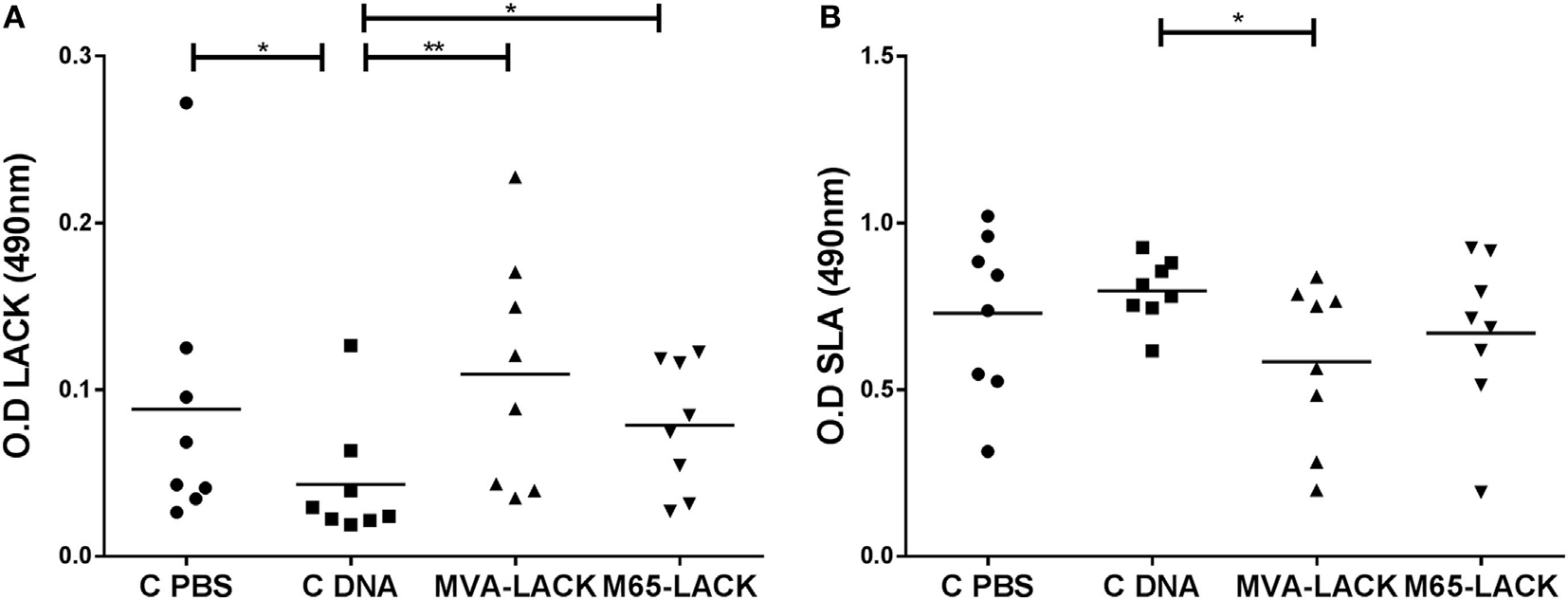
IgG levels against *Leishmania*-activated C-kinase antigen (LACK) **(A)** and soluble *Leishmania* antigen (SLA) **(B)** in the plasma of prime-boost immunized, C-DNA, and C-PBS group hamsters later infected with *Leshmania. infantum*. *p ≤ 0.05, **p ≤ 0.01.

The MVA-LACK and M65-LACK groups produced greater amounts of anti-IgG antibodies against LACK than did the C-DNA control group (*p* ≤ 0.01 and *p* ≤ 0.05, respectively) (Figure 2A). The C-PBS control group also produced greater amounts (*p* ≤ 0.05) of anti-LACK IgG antibodies than the C-DNA control group.

The MVA-LACK animals produced smaller amounts of anti-IgG antibodies against SLA than did the C-DNA control animals (*p* ≤ 0.05) (Figure 2B).

### After Stimulation With SLA, Both Vaccinated Groups of Hamsters Showed Increased PBMC Proliferation

**Figure 3:**
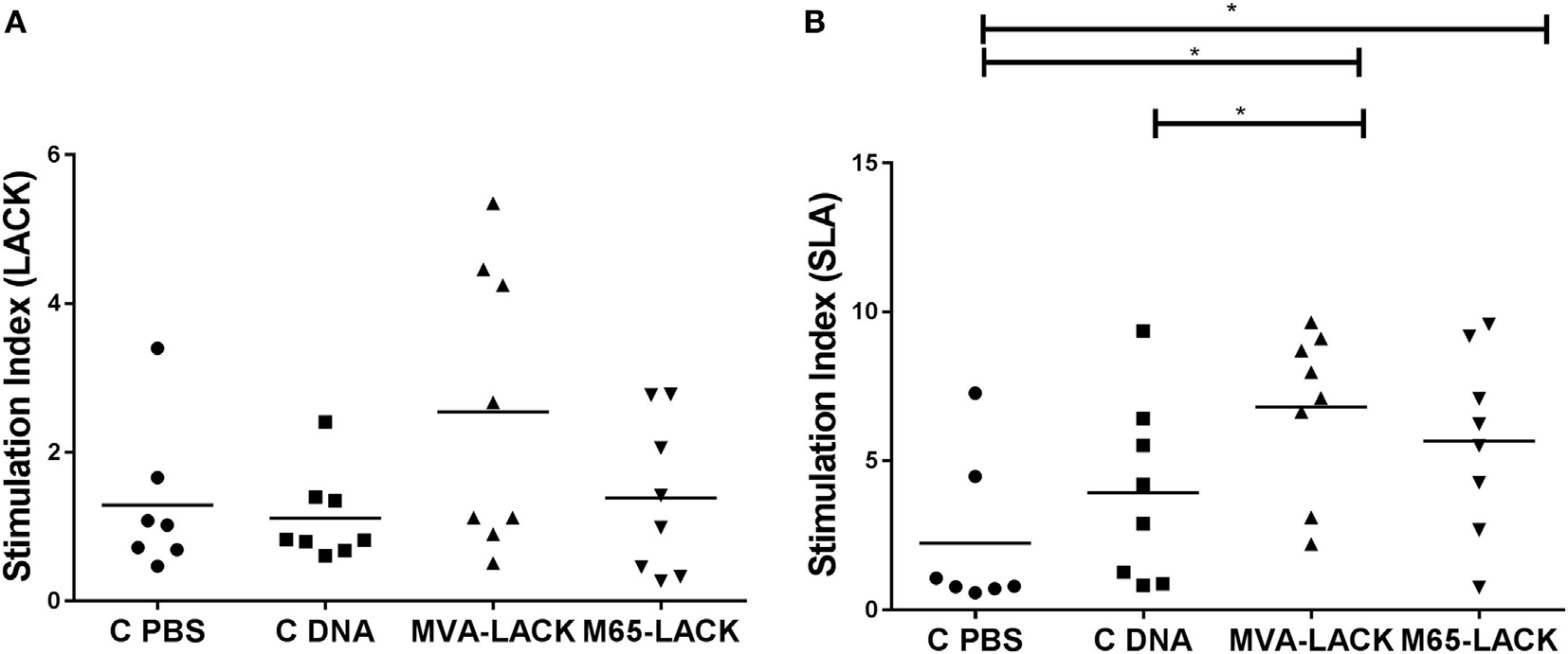
Post-infection peripheral blood mononuclear cells stimulation indices for C-PBS, C-DNA, and prime-boost immunized group hamsters in response to *Leishmania*-activated C-kinase antigen **(A)** and soluble *Leishmania* antigen **(B)**. *p ≤ 0.05.

Following stimulation with LACK, no differences were seen between any of the groups in terms of PBMC proliferation (Figure 3A). Lymphoproliferation after SLA stimulation, however, was significantly greater in the MVA-LACK group compared to the C-PBS and C-DNA controls (*p* ≤ 0.05). Proliferation was also greater in the M65-LACK group compared to the C-PBS group (*p* ≤ 0.05) (Figure 3B).

### Vaccinated Animals Showed Less Tissue Damage and Inflammation Than Control Group Animals

**Figure 4:**
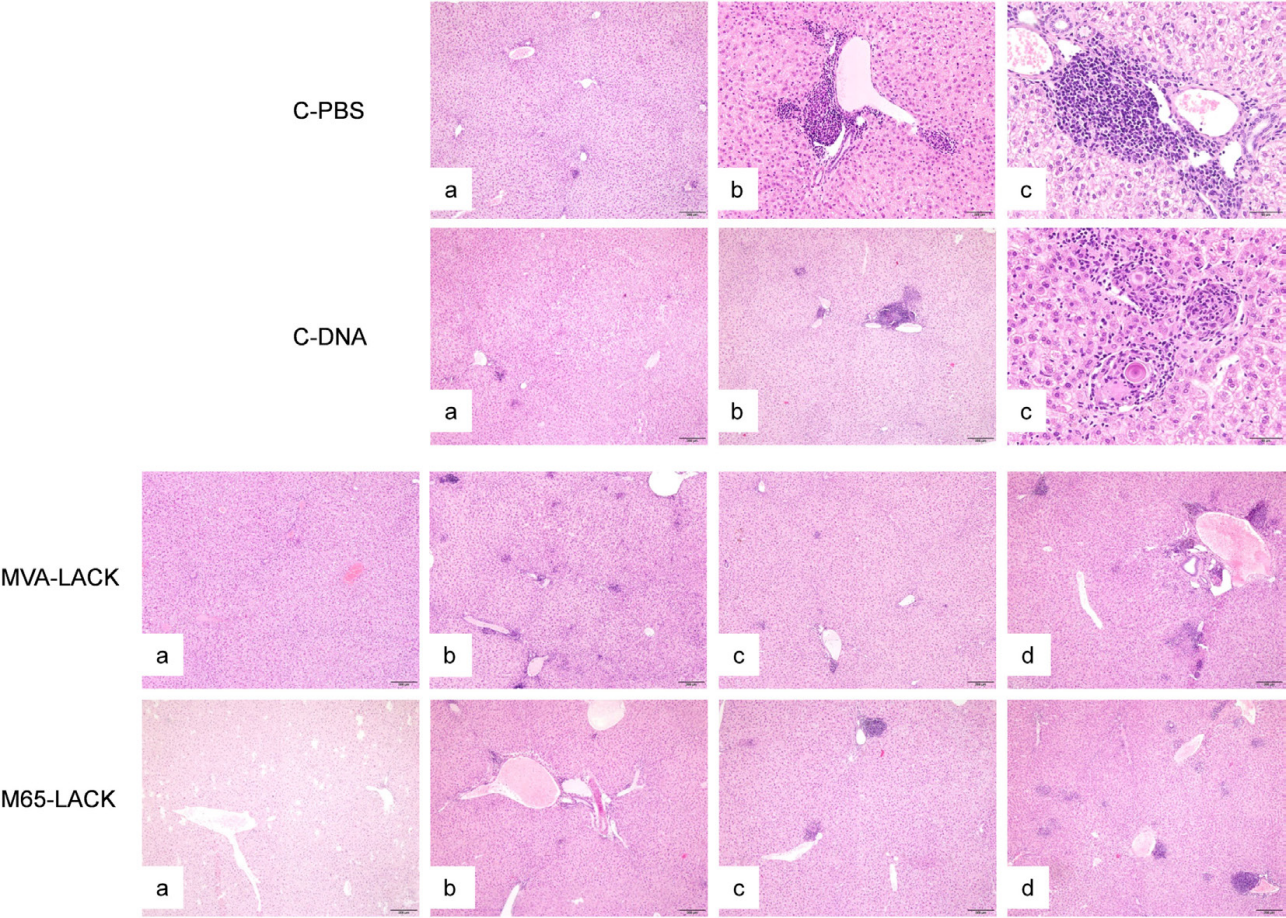
Liver histopathology of hamsters in the C-PBS, C-DNA, MVA-LACK, and M65-LACK groups. C-PBS group— mild, multifocal granulomatous hepatitis (a), moderate (b), and severe, multifocal, periportal, granulomatous hepatitis with intralesional Shauman bodies (c). C-DNA group—minimal hepatic inflammation (a), moderate multifocal, periportal granulomatous hepatitis (b), and severe, multifocal to coalescing granulomatous hepatitis with intralesional Shauman bodies (c). MVA-LACK group—minimal hepatic inflammation (a), mild, multifocal granulomatous hepatitis (b), moderate, multifocal, granulomatous hepatitis (c), and severe, multifocal to coalescing granulomatous hepatitis (d). M65-LACK group—minimal hepatic inflammation (a), mild, multifocal granulomatous hepatitis (b), moderate, multifocal granulomatous hepatitis (c), and severe, multifocal to coalescing, periportal, granulomatous hepatitis (d).

Inflammatory infiltrates were observed in the four groups and in all the examined organs with the exception of the kidneys. In the liver, inflammatory infiltrates were randomly distributed and varied in extension. The infiltrates were located surrounding portal triads and central veins. Inflammation was granulomatous in all cases, was frequently organized in granulomas, and consisted of macrophages, lymphocytes, plasma cells and neutrophils (Figure 4). Giant multinucleated cells and giant Langhans cells, many containing mineral concretions (Schauman bodies), were variably present regardless of the experimental group. Giant multinucleated cells, Langhans cells, and Schauman bodies were predominantly, though not exclusively, found in discrete granulomas (Figure 4). Amastigotes were very rarely observed within macrophages.

The spleen and bone marrow also contained variable infiltrates of granulomatous inflammation. No giant cells or Schauman bodies were observed in either organ in any of the groups.

The severity of the inflammation varied among the infected animals (Table 2). Animals in the PBS group had more severe granulomatous hepatitis than animals in the MVA-LACK group (Figure 4). The MVA-LACK group showed the least liver damage, followed by the M65-LACK group. A tendency to show inflammation in two (liver and spleen) or more organs was observed in both control groups.

**Table 2:** Histopathological findings.

Observations	Tissue	Groups			
		C PBS	C DNA	MVA-LACK	M65-LACK
Injuries severity	Liver	0 (0/8)	0 (2/8)	0 (2/8)	0 (1/8)
		+ (2/8)	+ (0/12)	+ (1/8)	+ (1/8)
		++ (2/8)	++ (2/8)	++ (3/8)	++ (4/8)
		+++ (4/8)	+++(4/8)	+++ (2/8)	+++ (2/8)

Multifocal to coalescing granulomatous infiltrates	Spleen	0 (6/8)	0 (4/8)	0 (6/8)	0 (5/8)
		+ (2/8)	+ (0/8)	+ (1/8)	+ (1/8)
		++ (0/8)	++ (3/8)	++ (1/8)	++ (2/8)
		+++ (0/8)	+++ (1/8)	+++ (0/8)	+++ (0/8)

General inflammation	Spleen	0 (6/8)	0 (4/8)	0 (6/8)	0 (5/8)
		1 (2/8)	1 (4/8)	1 (2/8)	1 (3/8)

*Severity of histopathological findings: non-existent (0), mild (+), moderate (++), severe (+++), and general inflammation (1)*.

*(n/n), number of animals with the given severity of the histopathological feature within each group*.

### Vaccination With pCI-neo-LACK/MVA-LACK and pCI-neo-LACK/M65-LACK Protected Hamsters Against *L. infantum*

**Figure 5:**
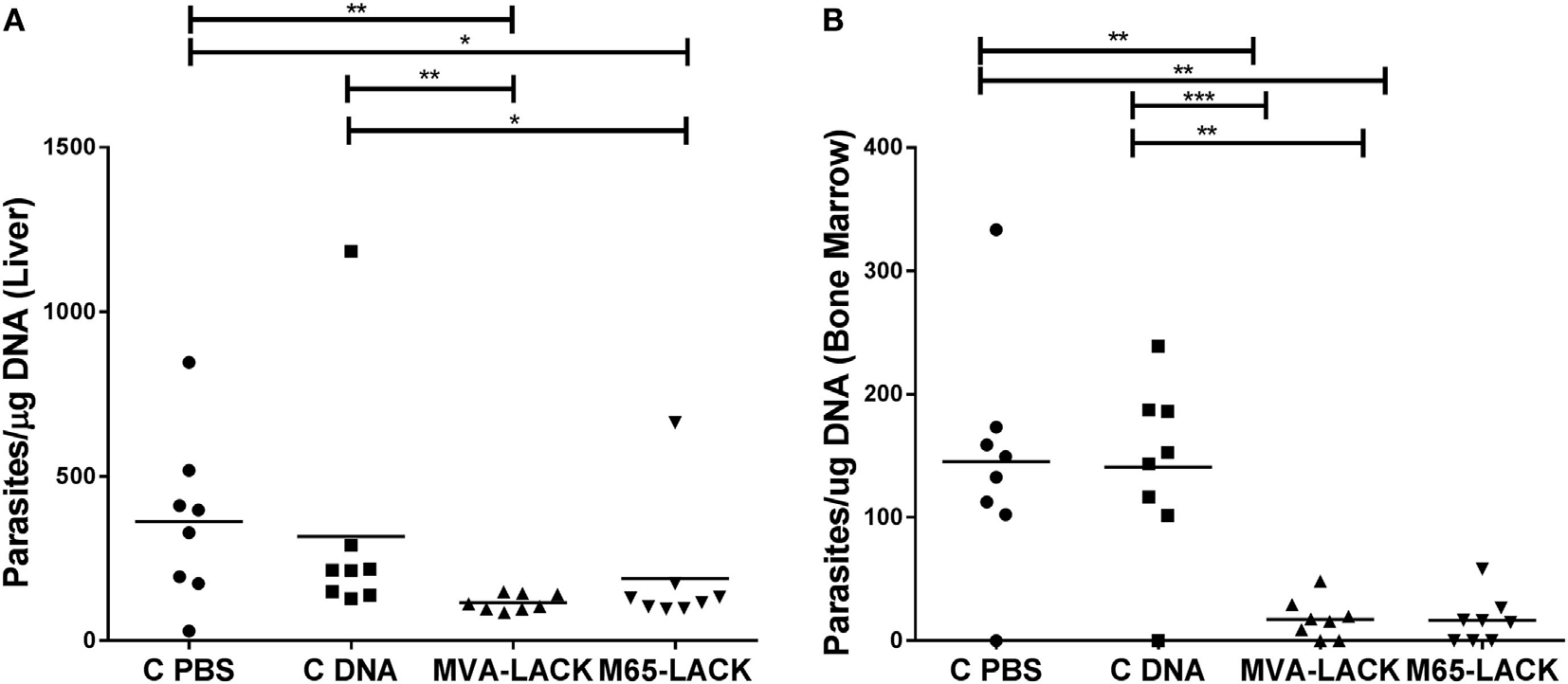
Liver and bone marrow amount of *Leishmania* DNA in the liver **(A)** and bone marrow **(B)** as determined by qPCR. *p ≤ 0.05, **p ≤ 0.01, ***p ≤ 0.001.

The animals of the MVA-LACK group showed a significantly smaller liver amount of *Leishmania* DNA than the C-PBS and C-DNA control groups (*p* ≤ 0.01), as did the M65-LACK animals (*p* ≤ 0.05) (Figure 5A). The MVA-LACK and M65-LACK treatments also led to significant reductions in the bone marrow amount of *Leishmania* DNA compared to the C-PBS (both *p* ≤ 0.01) and C-DNA control groups (*p* ≤ 0.001 and *p* ≤ 0.01, respectively) (Figure 5B). qPCR on spleen samples showed similar amount of *Leishmania* DNA among groups (data not shown).

## Discussion

The present results show that LACK recombinant protein is recognized by a high percentage of individuals with cellular immunity against the parasite, leading to the production of the Th1 cytokines (IFN-γ and TNF-α) and granzyme B. LACK protein is an immunodominant antigen of the parasite (12), that has previously been shown to induce the production of IFN-γ in lymphocytes from patients with active CL from *L. amazonensis, L. guyanensis*, and *L. braziliensis* (7, 12), as well as IFN-γ and TNF-α in asymptomatic individuals and patients cured of VL (23, 24). This is the first study in which PBMC from subjects cured of CL caused by *L. infantum* have been exposed to LACK. As in other groups of infected individuals, exposure to SLA and LACK elicited a Th1 response (production of IFN-γ and TNF-α) plus the production granzyme B. Although levels of LPS were residual (equal to 0.2 endotoxin units per mg protein), it is known that LPS-triggered cytokines produced by antigen-presenting myeloid cells contribute to the induction of T cell responses (25). For that reason, we cannot discard its potential adjuvant-like phenomenon in the present results (26).

Several studies have examined the capacity of *Leishmania* vaccine candidates to induce cytotoxic responses or to present epitopes restricted to HLA class I (9, 27–32). These showed that LACK-stimulation of PBMC induces the secretion of IFN-γ by CD8+ T cells in exposed individuals and patients with active CL (9, 31, 32). To our knowledge, this study is the first in which the LACK-induced production of granzyme B has been quantified in cured patients of CL and VL, and asymptomatic individuals from an *L. infantum*-endemic area. The cellular response seen in these subjects confirms that LACK induces specific cytotoxic responses in subjects with a cellular memory response against *L. infantum*.

Following LACK-stimulation, the PBMC of all individuals—including those of the control groups—produced IL-10, as previously reported (7, 12, 23, 24). This production has been linked to a dominant Th2-type epitope lying between amino acids 157 and 173 (FSPSLEHPIVVSGSWDN) (12) of the antigen.

The significant recognition of LACK by the T receptors of the present cured and asymptomatic subjects, plus the ability of this protein to induce IFN-γ, TNF-α, and granzyme B (CD4+ and CD8+ T cell responses), indicates this antigen to be of potential use in vaccines against human leishmaniasis. However, the intrinsic ability of LACK to also induce IL-10 production and to expand IL-4-secreting T cells (13) illustrates the need to further optimize a LACK-based vaccine for the induction of a protective Th1 response. DNA vaccines involving the LACK antigen have returned promising results in murine models of *L. major* and *L. amazonensis* infection, as well as in a canine model of *L. infantum* infection (8, 17, 18, 33). In these earlier studies, the vaccines were applied under a heterologous immunization protocol, stimulating humoral, Th1-type, and cytotoxic immune responses.

The relevant effect observed in human PBMC from asymptomatic and cured patients of increase secretion of IFN-gamma and granzyme B in response to LACK, provided the rational to explore the significance of these findings in the hamster model of VL. Both the DNA-LACK/MVA-LACK and DNA-LACK/M65-LACK vaccines induced the production of anti-LACK antibodies beyond that seen in the C-DNA group. Immunization with both vaccines also induced a potent cellular response, as shown by the higher mean stimulation index to SLA observed in the immunized groups after experimental infection with *L. infantum* promastigotes. Vaccinia viruses are capable of inducing CD4+ and CD8+ T cell responses. In a mouse CL model, heterologous immunization with DNA-LACK/MVA-LACK has been reported to preferentially promote a CD8+ T cell-type response, whereas that generated by DNA-LACK/M65-LACK immunization is preferably of the CD4+ T cell type (17). In the present efficacy trial in hamsters, a similar immune response was observed in both the MVA-LACK and M65-LACK groups. As seen for the present response against VL in hamsters, Ramos et al. reported that dogs vaccinated with DNA-LACK/MVA-LACK showed greater lymphoproliferation after SLA-stimulation than did control animals. This specific immune response was related to the protection conferred by the vaccine in the canine model of *L. infantum* infection (18). In our study, the patent cellular response induced by both DNA-LACK/MVA-LACK and DNA-LACK/M65-LACK was associated with the control of the amount of *Leishmania* DNA observed in the liver and bone marrow. The extension and severity of the inflammatory infiltrates in the liver, observed histologically were not significant between groups, however. This could in part be explained because MVA-LACK and M65-LACK induce a more effective cellular response, as suggested by the higher lymphoproliferation values and lower parasite burdens, rather than a reduction in the quantity of the cellular response to the infection.

However, other authors report that immunization with plasmid DNA expressing the LACK antigen fails to induce any protection in mice infected with *L. infantum* or *L. donovani* (34, 35). The discrepancy with the present results is likely due to the fact that these earlier studies involved only homologous immunization with a LACK-expressing plasmid, while our studies were performed with heterologous combination of vectors, a protocol known to activate strong B and T cell immune responses to LACK antigen (31).

In conclusion, LACK is well recognized by the T cells from individuals cured of leishmaniasis, and by those of asymptomatic subjects living in a *L. infantum*-endemic area. Prime-boost vaccination with pCI-neo-LACK/MVA-LACK, and pCI-neo-LACK/M65-LACK, protected against *L. infantum* infection in a hamster model of VL. These results highlight the significance of LACK as immune activator and suggest that LACK should be considered in the formulation of a vaccine designed to induce a cellular response against VL in humans.

## Ethics Statement

Work involving human subjects was approved by the Hospital de Fuenlabrada Ethics and Research Committee (APR 12-65 and APR 14-64). All participants gave their written, informed consent to be included. Work involving animals was approved by the Research Ethics and Animal Welfare Committee of the Instituto de Salud Carlos III, and performed adhering to Spanish legislation on the protection of animals used for experimentation and other scientific purposes (Royal Decree 1201/2005 and Law 32/2007; this law is a transposition of Directive 86/609/EEC).

## Author Contributions

LF, LS-S, CS, AI-M, MJ, and VA conducted the experiments. LF, CS, and AI-M acquired the data. LF, EC, MJ, and VA analyzed the data. EC, LS-S, ME, and JM designed the research studies. EC, LS-S, ME, and JM provided reagents. LF, EC, and JM wrote the manuscript. All authors have read and approved the final manuscript.

## Conflict of Interest Statement

The authors declare that the research was conducted in the absence of any commercial or financial relationships that could be construed as a potential conflict of interest.
